# Supplementary material for: An Open-Source Photogrammetry Workflow for Reconstructing 3D Models
Source: Integr Org Biol. 2023 Jul 7;5(1):obad024. doi: 10.1093/iob/obad024 (PMC10350669; doi:10.1093/iob/obad024)
Supplement: obad024_Supplemental_Files [file obad024_supplemental_files.zip › supplement_workflow instructions.docx]

**Photogrammetry workflow instructions:**

Step-by-step instructions on photogrammetry workflow from setup to photography to 3D model reconstruction and data visualization are provided in detail at our online repository hosted on the OSF.io website:

<https://doi.org/10.17605/OSF.IO/B39YX>

We refer the readers to use this repository for most up-to-date instructions, along with sample datasets, 3D model outputs and landmarks associated with the study.

To access and download the step-by-step instructions from the OSF.io website, click “the ODM Photogrammetry Instructions.docx” and then download the file (marked in red).


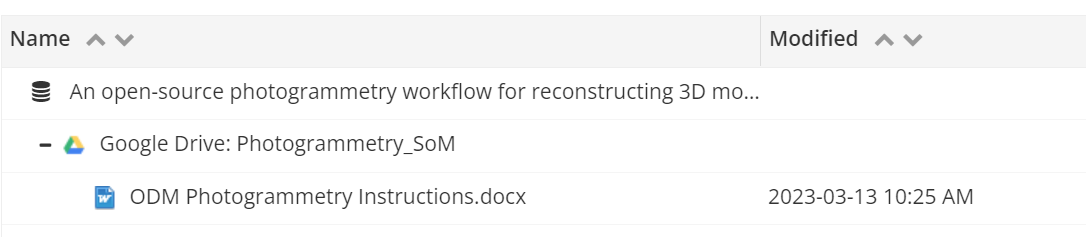


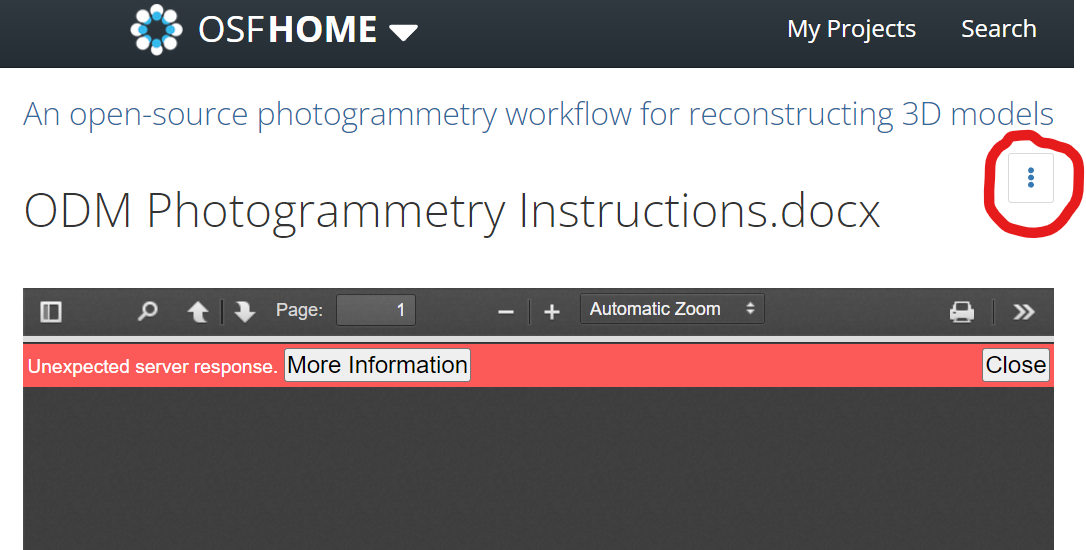


The instructions can also be accessed at: <https://docs.google.com/document/d/1YP72Q90UbsUUryi3Yeu8ccRSJuibTQni/edit?usp=sharing&ouid=111985310108033478733&rtpof=true&sd=true>

**Prerequisites: Setting ODM and finding help:**

There are multiple ways ODM and WebODM can be installed. We opted to use the docker-based installation of the WebODM on our cloud server as it is the simplest to start. <Please> follow the instruction on setting up the WebODM at: <https://github.com/OpenDroneMap/WebODM/#getting-started>

The official documentation and tutorials of ODM (and WebODM) can be found at: <https://docs.opendronemap.org/>.

The user community form of ODM is at: <https://community.opendronemap.org/>

The postprocessing of the textured model and landmark data collection requires MeshLab and 3D Slicer with the SlicerMorph extension. MeshLab can be downloaded from <https://www.meshlab.net/>. For installing 3D Slicer and SlicerMorph, please see <https://github.com/SlicerMorph/SlicerMorph#installation>

A python programming environment is necessary if the user wants to scale the specimen using Aruco markers.

**Suggested equipment and supplies:**

- A DSLR camera (Canon EOS Rebel T6)
- A programmable turntable that can be controlled by a remote controller to set up how many steps it takes to finish a full circle and can be synced with a camera (The turntable we used was ComXim 12.6inch turntable with a shutter cable)
- Portable photo studio box (Amazon Basics) and lighting system (two UBeeSize V107 lights)
- Tripod (Amazon Basics)
- Putty
- A vertical stand for fixing the putty
- A remote shutter cable to connect the turntable with the camera, and a USB cable to connect the camera to the computers
